# 

**Published:** 1856-05

**Authors:** 


### CONTENTS.

ORIGINAL COMMUNICATIONS— PAGE.

Valedectory Address to the Graduates of Rush Medical College, Session 1855–6. By W. B. Herrick, M.D., Professor of Physiology and Pathology, ........ 199

Pre-expulsion of the Placenta in Child-birth. By C. Goodbrake, of Clinton, Illinois, ........ 211

Neuro-Pathology of Intermittent Fever. By G. W. Hall, M.D., of Bainbridge, Indiana, ........ 213

Fœtal Malformation. By W. W. Adams, M.D., of Clinton, Ill., ........ 215

SELECTIONS—

Lectures on Experimental Physiology, delivered at the College de France, Paris, during the year 1855. By Professor Claude Bernard. From Notes taken at the Lectures by Anthony Peniston, M.D., of New Orleans, ........ 218

BOOK NOTICES, ........ 231

EDITORIAL, ........ 238

